# A Systematic Review and Meta-Analysis of the Association between Water, Sanitation, and Hygiene Exposures and Cholera in Case–Control Studies

**DOI:** 10.4269/ajtmh.17-0897

**Published:** 2018-07-02

**Authors:** Marlene Wolfe, Mehar Kaur, Travis Yates, Mark Woodin, Daniele Lantagne

**Affiliations:** Tufts University Civil and Environmental Engineering Department, Medford, Massachusetts

## Abstract

Case–control studies are conducted to identify cholera transmission routes. Water, sanitation, and hygiene (WASH) exposures can facilitate cholera transmission (risk factors) or interrupt transmission (protective factors). To our knowledge, the association between WASH exposures and cholera from case–control studies has not been systematically analyzed. A systematic review was completed to close this gap, including describing the theory of risk and protection, developing inclusion criteria, searching and selecting studies, assessing quality of evidence, and summarizing associations between cholera and seven predicted WASH protective factors and eight predicted WASH risk factors using meta-analysis and sensitivity analysis. Overall, 47 articles describing 51 individual studies from 30 countries met the inclusion criteria. All eight predicted risk factors were associated with higher odds of cholera (odds ratio [OR] = 1.9–5.6), with heterogeneity (*I*^2^) of 0–92%. Of the predicted protective factors, five of seven were associated with lower odds of cholera (OR = 0.35–1.4), with heterogeneity of 57–91%; exceptions were insignificant associations for improved water source (OR = 1.1, heterogeneity 91%) and improved sanitation (OR = 1.4, heterogeneity 68%). Results were robust; 3/70 (5%) associations changed directionality or significance in sensitivity analysis. Meta-analysis results highlight that predicted risk factors are associated with cholera; however, predicted protective factors are not as consistently protective. This variable protection is attributed to 1) cholera transmission via multiple routes and 2) WASH intervention implementation quality variation. Water, sanitation, and hygiene interventions should address multiple transmission routes and be well implemented, according to international guidance, to ensure that field effectiveness matches theoretical efficacy. In addition, future case–control studies should detail WASH characteristics to contextualize results.

## INTRODUCTION

Cholera is an acute, diarrheal disease caused by toxigenic *Vibrio cholerae*.^1^ Cholera can be endemic or epidemic, and has an estimated global burden of 1.4–4.3 million cases and 28,000–142,000 deaths annually.^2^ Since its appearance in Asia in 1817, there have been seven cholera pandemics, with the most recent occurring from 1961 until the present and reaching Africa and the Americas.^1,3^ Cholera spreads through the fecal–oral route, via ingestion of fecally contaminated water and food containing the cholera bacteria.^4,5^

**Figure 1.**
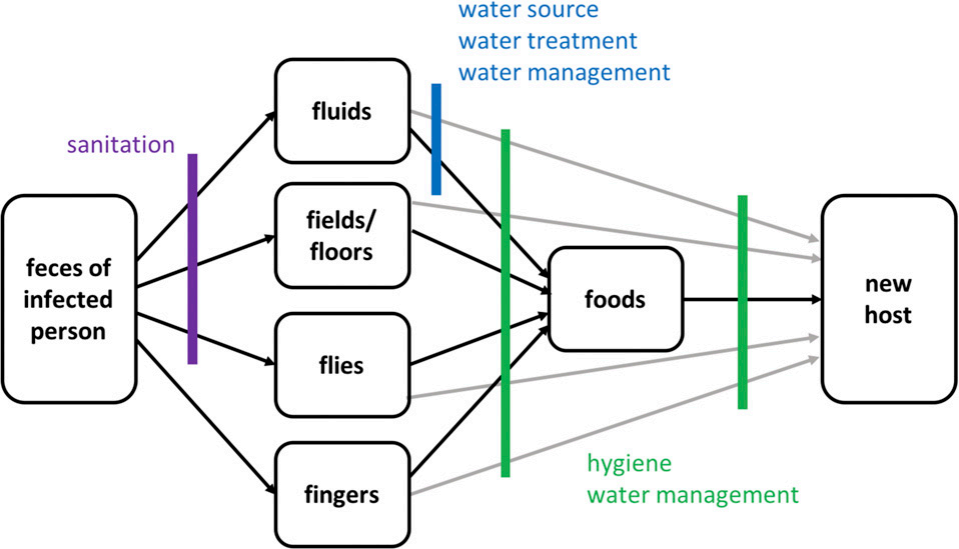
F-diagram showing pathways of fecal–oral disease and opportunities to interrupt transmission.^7^ This figure appears in color at www.ajtmh.org.

Water, sanitation, and hygiene (WASH) interventions are commonly implemented to prevent and control cholera by blocking exposures assumed to be risk factors for disease transmission (Figure 1).^6^ Water interventions improve the quantity of water (e.g., water trucking), the quality of water (e.g., chlorinating water), or the management of water (e.g., safe storage). Sanitation interventions separate feces from the environment by providing facilities and/or proper waste disposal (e.g., building a latrine) and hygiene interventions prevent transmission by cleaning oneself or the home environment (e.g., handwashing with soap). Water, sanitation, and hygiene exposures that are predicted to interrupt cholera transmission are termed “protective factors” (e.g., safe drinking water) and those that are predicted to facilitate transmission are “risk factors” (e.g., open defecation).

In 2017, to fill an identified evidence gap,^8–11^ a systematic review of the efficacy and effectiveness of WASH interventions in outbreaks was completed by Yates et al.^6^ Only six health impact evaluations were identified, with all six evaluations documenting reduced disease rates. More commonly, evaluations documented reductions of transmission risk, such as chlorine residual presence in household drinking water. Simple WASH interventions that were appropriately timed, community-driven, and had linkages between relief and development were found to be most effective. Taste and smell, communication methods, inaccurate perception of efficacy, and trust/fear were consistently found to influence program success. Overall, WASH interventions reduced both the risk of disease and the risk of disease transmission in outbreaks; however, program design and beneficiary preferences were important considerations to ensure WASH intervention field effectiveness matched efficacy.

A limitation of the Yates review is that case–control studies were not included, as the methodology to assess case–control studies is different from that of other study types that were included, which were population-based. Case–control studies are observational studies in which individuals with a disease (cases) are recruited along with individuals who have not had the disease (controls). Exposures are retrospectively compared to determine how frequently various exposures are present in each group.^12^ Case–control studies are completed because they are inexpensive, rapid, cross-sectional, and provide evidence on whether exposures are protective factors or risk factors. Although they cannot determine causality, case–control studies provide some of the best evidence on health in emergencies and outbreaks because they are relatively easy to conduct even in a crisis and can yield information about the sources of the outbreak that can then be used to develop response activities.

To our knowledge, the evidence on the association between WASH exposures and cholera transmission has not been summarized from case–control studies. To fill this evidence gap, we performed a systematic review of cholera case–control studies to summarize the association between WASH exposures and cholera transmission.

## METHODS

We conducted a systematic review of published literature to evaluate the association between WASH exposures and cholera transmission. The review was developed based on the guidelines for the Preferred Reporting Items for Systematic Reviews and Meta-Analyses^13^ and included the development of 1) a definition of risk and protection, 2) a search strategy, 3) the inclusion criteria, 4) a selection and data extraction strategy, 5) the framework for appraising risk of bias, and 6) an analysis plan. Each of these steps is described in the following paragraphs.

### Theory of risk and protection.

A priori, and based on the F-diagram (Figure 1), five WASH groups that theoretically impact cholera transmission were defined, including water source, water treatment, water management, sanitation, and hygiene. During data extraction, all individual WASH exposures detailed in included case-control studies were categorized into one of these five WASH groups (e.g., the exposure “chlorinated drinking water” was categorized into the WASH group “water treatment”). Definitions for exposures in each of these categories are based on the United Nations Children’s Fund/World Health Organization (WHO) Joint Monitoring Program (JMP) standards.

During analysis, the exposures included within each WASH group were subdivided into predicted protective factors and predicted risk factors, based on the F-diagram. Note that data are presented herein for predicted protective and risk factors with at least five exposures from case–control studies in at least three included articles. Because exposures were chosen for inclusion based on the presence of data among the selected studies, the theory of risk and protection for each predicted protective and risk factor is specifically described in the Results section.

### Search strategy.

In July 2016, the databases Web of Science and Medline (Pubmed) were searched using the following string: (“case control” OR “case-control” AND “cholera”). Because WASH exposures are often evaluated but not highlighted in the abstracts of these articles, the search was kept intentionally broad without reference to WASH exposures in the search string. The search was limited to peer-reviewed English-language articles published from 1990. References were stored in Zotero 4.029.15 (Corporation for Digital Scholarship and Roy Rosenzweig Center for History and New Media, Fairfax, VA), and duplicates deleted.

### Inclusion criteria.

Inclusion criteria were defined according to the populations, interventions, comparisons, outcomes, and study type (PICOS) framework, a model recommended by the Cochrane Library to structure rigorous reviews on health-related questions.^14^

#### Populations.

Populations included in the review must have been affected by cholera. All age, gender, and socioeconomic populations in cholera were included.

#### Interventions/exposures.

Because this review is based on case–control studies, we define studies evaluating eligible exposures, rather than interventions. Although we were not able to evaluate interventions, protective exposures represent interventions that should limit cholera transmission. Studies were eligible for inclusion if they included exposures from one of the five WASH groups identified in the theory of risk and protection development: water source, water treatment, water management, sanitation, and hygiene. Studies were excluded if they were designed to evaluate a cholera vaccine program, as the interaction between WASH exposures and vaccination is currently unknown.^15^

#### Comparisons.

Specific comparisons were not required for inclusion.

#### Outcomes.

Studies were eligible for inclusion if they reported an association between cholera and at least one WASH exposure using an odds ratio (OR).

#### Study types.

Only case–control studies were eligible for review.

### Selection and data extraction.

Studies were screened by two independent authors in each of the two screening stages: Screening 1) articles were excluded if the outcome was not cholera cases or the study design was not case–control in title and abstract screening and Screening 2) the full text of the articles selected in Screening one was examined and studies that did not meet the aforementioned PICOS criteria were excluded. Discrepancies between reviewers were resolved through discussion and consensus.

Relevant data were extracted from each article according to the framework in Waddington et al.,^16^ including author and publication details, WASH exposures, study design features (e.g., matching of cases and controls), case and control definitions, number of cases and controls, geographic region, and demographic information.^16^ Quantitative data extracted included sample size and impact estimates (ORs) and 95% confidence intervals (CIs) for each exposure. Individual WASH exposures were grouped into WASH-predicted protective factors and predicted risk factors as described earlier in the theory of risk and protection section. In addition, case definitions used in the individual studies were examined to determine if they matched the WHO cholera case definition of: “severe dehydration or death from acute watery diarrhea in an individual 5 years or older in an area where the disease is not known to be present, or an individual 5 years or older who develops acute watery diarrhea, with or without vomiting, in an area where the disease is known to be present.”^17^ Data were managed using a coding sheet developed in Microsoft Excel 2010 (Microsoft Corp., Redmond, WA) and were coded by two independent reviewers.

### Risk of bias appraisal.

A quality assessment tool adapted from the Quality Assessment Tool for Quantitative Studies by the Effective Public Health Practice Project and Baird et al.’s (2013) version of the *Cochrane Handbook* “Risk of Bias” tool was used to assess risk of bias in included studies.^18,19^ These tools were adapted to apply specifically to case–control studies by removing the criteria that referenced intervention implementation and long-term follow-up. The risk of bias was assessed across five categories: 1) selection and confounding, 2) spillover and contamination, 3) incomplete outcomes, 4) selective reporting, and 5) other bias. Each study was scored as “Low Risk,” “High Risk,” or “Unclear” for each of these categories, and these determinations were used to generate a summary of risk bias. Studies scoring “Low Risk” in 4–5 categories were determined to have an overall low risk of bias, those with 3 “Low Risk” scores were determined to have a medium risk of bias, and those with 1–2 “Low Risk” scores were determined to have a high risk of bias.

### Analysis.

Data management and analysis were performed in Microsoft Excel and Stata 14 (StataCorp LP, College Station, TX). Meta-analyses were performed in Stata 14 using the ORs associated with exposures for each of the 15 factors (seven predicted protective factors and eight predicted risk factors) to determine an overall association. A summary OR was generated for each of these 15 factors using a Mantel–Haenszel random effects analysis. Random effects analysis was used because of the high heterogeneity that was observed between estimates from case–control studies. An *I*^2^ test was used to formally quantify the amount of statistical heterogeneity observed, with significance determined using a Pearson χ^2^ test. Despite heterogeneity between studies, meta-analysis was deemed appropriate to use because of the universality of fecal exposure risk in transmitting cholera.

To assess the robustness of the overall associations, five sensitivity analyses were completed, by performing the same analysis by factors including only exposures from 1) studies assessed as low and medium risk of bias, 2) studies assessed as low risk of bias, 3) studies of any risk level that used the WHO case definition for cholera, 4) low- and medium-risk studies that used the WHO case definition, and 5) low-risk studies using the WHO case definition. Sensitivity analysis is reported in the main text when the association changed direction or became either newly significant or insignificant.

## RESULTS

**Figure 2.**
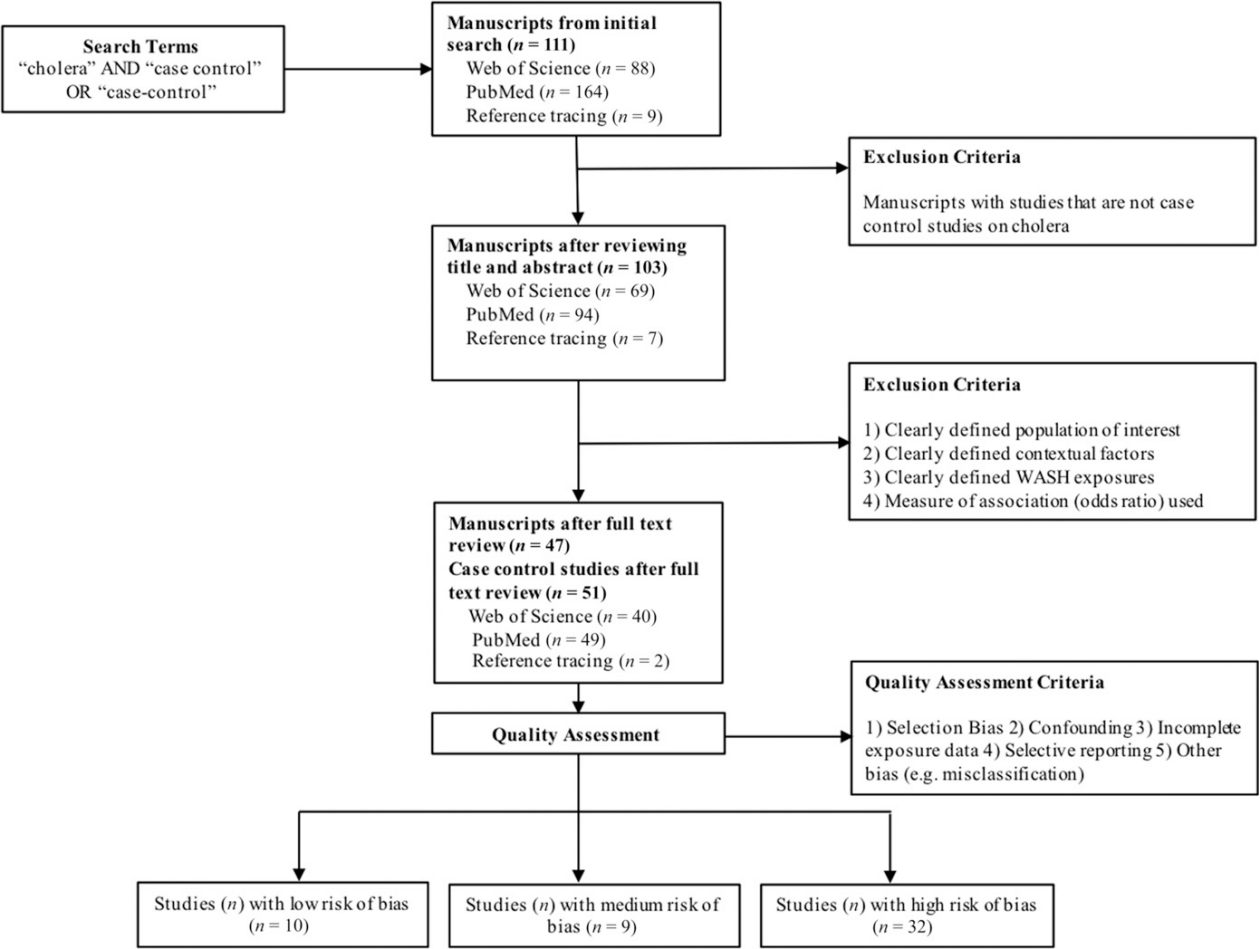
Study selection and quality assessment flow chart.

Overall, 111 articles were identified in the initial search, 103 articles were included after reviewing title and abstract in the first screening, and 47 articles, including 51 individual case–control studies, were included after full-text review in the second screening (Supplemental Table 1, Figure 2). The articles represent studies from 30 countries; five were from Kenya, followed by four each from India, Haiti, Papua New Guinea, and Malawi. In the quality assessment, 10 studies (20%) were categorized as having low risk of bias, nine (18%) as medium risk of bias, and 32 (63%) as high risk of bias. Of 51 studies, 26 (51%) used the WHO definition of cholera. In total, 15 factors were found to fall under the five WASH groups defined as impacting cholera transmission, including seven predicted protective factors and eight predicted risk factors (Table 1).

**Table 1:** Predicted protective and risk factors, by WASH group

WASH group	Predicted protective factors	Predicted risk factors
Water source	Improved water source	Unimproved water source
	Bottled water source	Surface water contact
Water treatment	Treated water	Untreated water
Water management	Safe water storage and transport	Unsafe water storage and transport
Sanitation	Improved sanitation	Open defecation
		Unimproved sanitation
		Shared sanitation
Hygiene	Self-report good hygiene	Self-reported lack of hygiene
	Observation of hygiene materials	

WASH = water, sanitation, and hygiene.

### Water source.

Predicted protective factors (improved water source and bottled water source) and predicted risk factors (unimproved water source and surface water contact) were developed based on exposures identified in the review. Improved water source was defined according to JMP standards and includes sources that adequately protect water from outside contamination such as piped water, boreholes, protected springs, and rainwater.^20^ The use of an improved water source is a predicted protective factor because protection from outside contamination is a barrier to fecal–oral contamination (Figure 1).^21,22^ Bottled water is classified by the JMP as improved but was assessed separately from improved water, also as a predicted protective factor, because bottled water was recently reclassified by the JMP from unimproved to improved.^20^ Unimproved water does not provide protection from contamination and was, therefore, a predicted risk factor. Contact with surface water was reported in included studies as an exposure and a predicted risk factor, as surface water is considered unimproved by the JMP.

**Figure 3.**
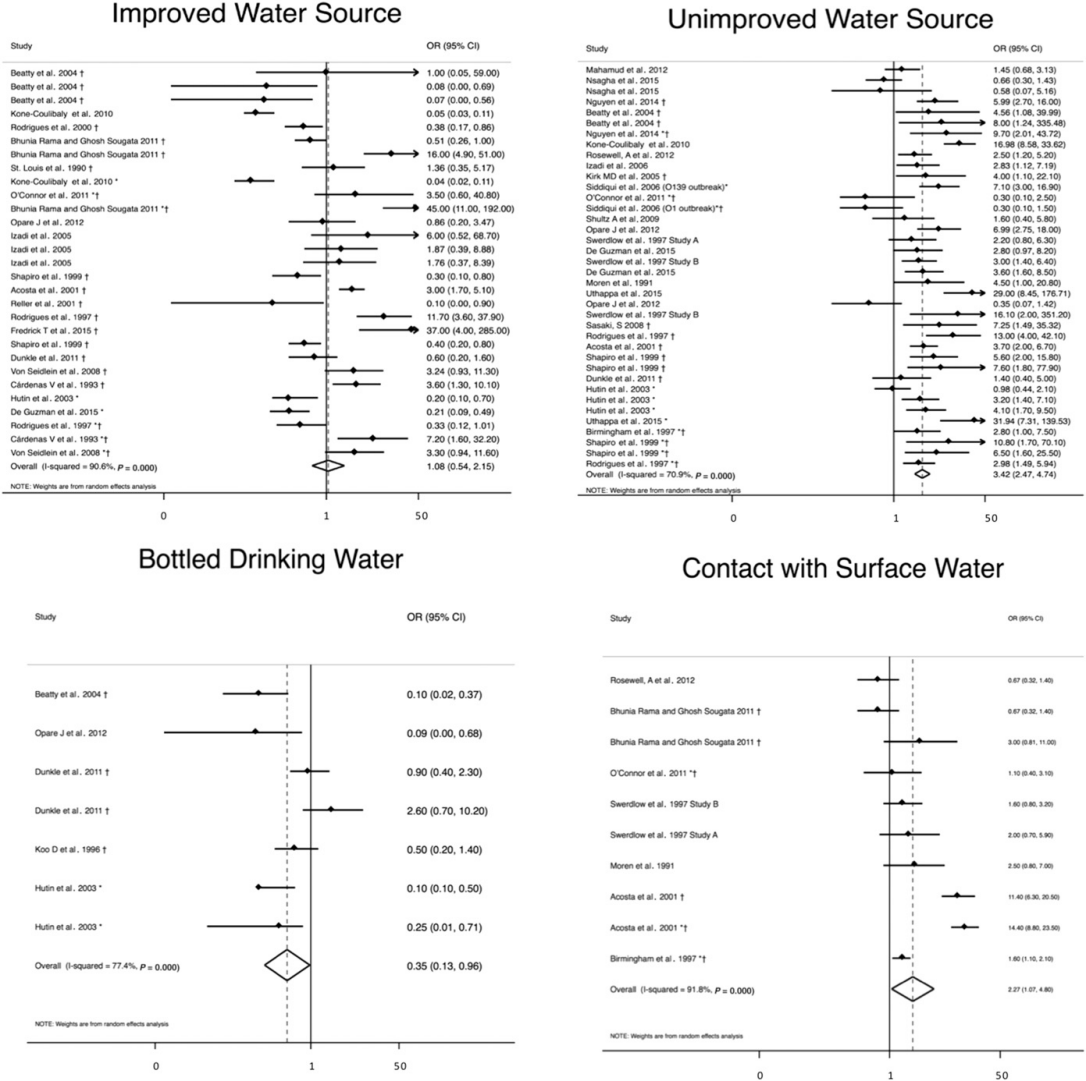
Meta-analysis of the association between water source and cholera, including improved and unimproved water sources, bottled drinking water, and contact with surface water. *Odds ratio (OR) reported from multivariate analysis. †Study used the WHO case definition for cholera.

Improved water source (*N* = 28) was not associated with significantly lower odds of cholera (OR = 1.1, 95% CI = 0.54–2.2) with a heterogeneity of *I*^2^ = 91%,^23–40^ but bottled drinking water was (*N* = 7, OR = 0.35, 95% CI = 0.13–0.96) with a heterogeneity of *I*^2^ = 77% (Table 2, Figure 3).^23,29,32,39,41^ The use of an unimproved water source (*N* = 38) was significantly associated with higher odds of cholera (OR = 3.4, 95% CI = 2.5–4.7) with a heterogeneity of *I*^2^ = 71%.^23,24,28–30,32,35,36,38–40,42–52^ Surface water contact (*N* = 10) was significantly associated with higher odds of cholera (OR = 2.3, 95% CI = 1.1–4.8) with a heterogeneity of *I*^2^ = 92%).^25,28,30,45,48,49,52^

**Table 2:** ORs and 95% CIs from predicted protective and risk factors

Predicted protective factors	*N* (exposures)	OR (95% CI)	*I*^2^	Predicted risk factors	*N* (exposures)	OR (95% CI)	*I*^2^
Improved water source	28	1.08 (0.54–2.15)	91%*	Unimproved water source	38	3.42 (2.47–4.74)	71%*
Bottled water source	7	0.35 (0.13–0.96)	77%*	Surface water contact	10	2.27 (1.07–4.80)	92%*
Treated water	40	0.44 (0.35–0.56)	61%*	Untreated water	34	3.47 (2.76–4.35)	48%*
Safe water storage and transport	22	0.55 (0.39–0.80)	57%*	Unsafe water storage and transport	22	2.79 (2.13–3.65)	45%*
Improved sanitation	16	1.37 (0.90–2.10)	68%*	Open defecation	7	5.62 (3.45–9.14)	0%
Self-reported good hygiene	39	0.35 (0.27–0.45)	65%*	Unimproved sanitation	13	2.46 (1.22–4.94)	76%*
Observation of hygiene materials	28	0.34 (0.23–0.49)	67%*	Shared sanitation	14	1.90 (1.49–2.43)	0%
–	–	–	–	Self-reported lack of hygiene	11	3.75 (2.44–5.77)	43%

CI = confidence interval; OR = odds ratio.

* Heterogeneity significant at the *P* = 0.05 level (*I*^2^ test of heterogeneity with significance determined with Pearson’s χ^2^ test).

In sensitivity analysis, unimproved water source became insignificantly associated with cholera when only studies with low- and medium-bias risk using the WHO case definition (*N* = 21) were included (OR = 2.4, 95% CI = 0.78–7.7) (Supplemental Table 2).

### Water treatment.

Water treatment was defined as measures taken to make water safer to drink, including boiling, filtering, or treating with chlorine. Water treatment was a predicted protective factor, as these interventions are intended to remove or inactivate bacteria introduced through fecal–oral contamination.^53–55^ Lack of treatment allows water to remain contaminated and can contribute to the spread of disease, and was a predicted risk factor.

**Figure 4.**
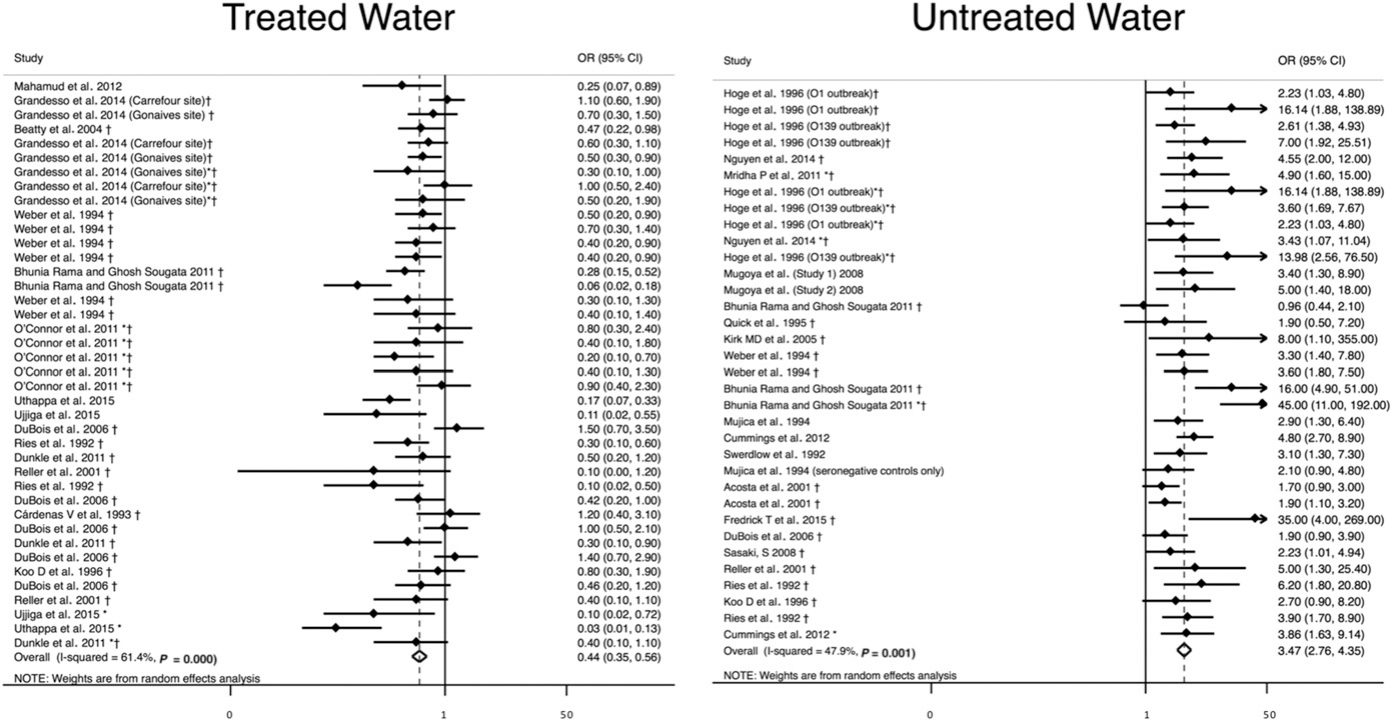
Meta-analysis of the association between water treatment and cholera, including water treatment and no water treatment. *Odds ratio (OR) reported from multivariate analysis. †Study used the WHO case definition for cholera.

Untreated water (*N* = 34) was significantly associated with higher odds of cholera (OR = 3.5, 95% CI = 2.8–4.4) with a heterogeneity of *I*^2^ = 48%,^25,30,33,34,41,44,46,51,56–65^ and water treatment (*N* = 40) was significantly associated with lower odds of cholera (OR = 0.44, 95% CI = 0.35–0.56) with a heterogeneity of *I*^2^ = 61% (Table 2, Figure 4).^23,25,28,31,32,34,41,42,50,60,64–67^

There were no changes in direction of association or significance in sensitivity analysis (Supplemental Table 2).

When looking at types of water treatment, boiling (*N* = 13) was associated with lower odds of cholera (OR = 0.35, 95% CI = 0.24–051) with a heterogeneity of *I*^2^ = 58%,^25,31,34,41,50,60,64,65^ as was treatment with chlorine (*N* = 17, OR = 0.58, 95% CI = 0.41–082) with a heterogeneity of *I*^2^ = 60%.^25,28,32,34,64,66^ Chlorination of water that was confirmed via free chlorine residual testing (*N* = 7) was not associated with cholera (OR = 0.88, 95% CI = 0.63–1.2) with a heterogeneity of *I*^2^ = 3.8%.^28,64,66^

### Water management.

Water management includes practices related to water transport and storage. Safe water transport and storage prevents contamination of water through the use of protected containers such as covered buckets and jerricans, and constitutes a barrier to fecal-oral contamination.^55,68^ Safe practices were predicted protective factors. Unsafe water management practices include transporting or storing water in a container with no lid, and were predicted risk factors as they allow for contamination of water.

**Figure 5.**
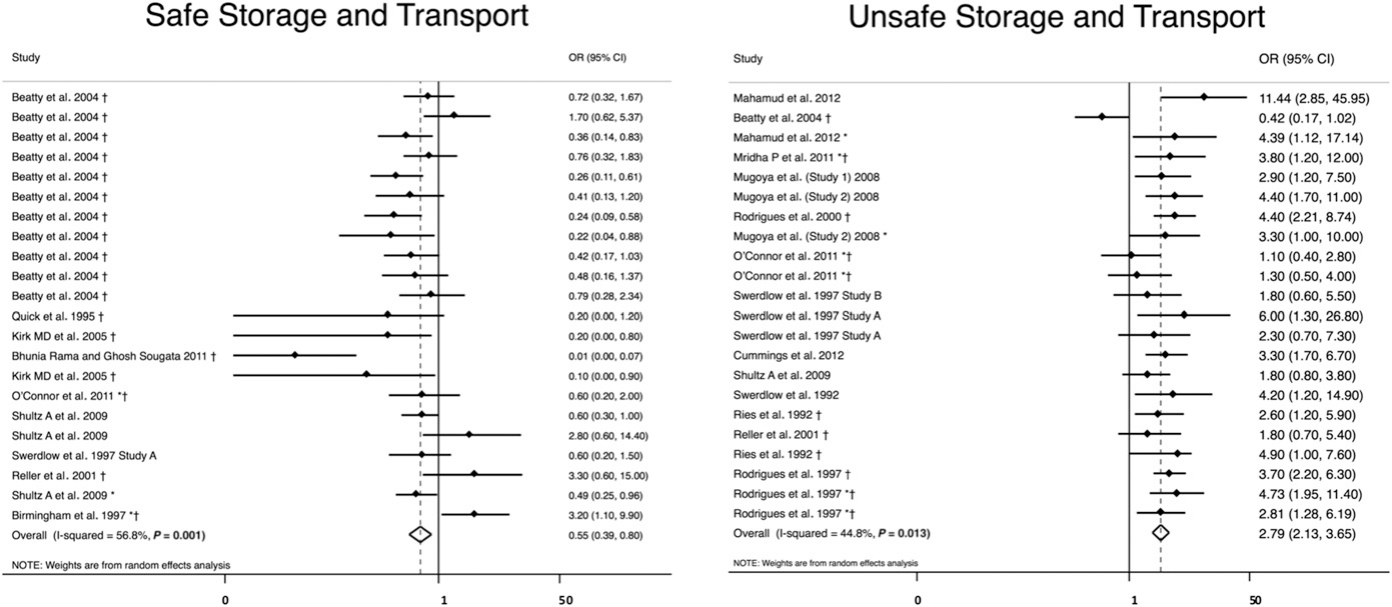
Meta-analysis of the association between methods of water transport and storage and cholera, including safe water transport and storage and unsafe transport and storage. *Odds ratio (OR) reported from multivariate analysis. †Study used the WHO case definition for cholera.

Unsafe water transport and storage (*N* = 22) was significantly associated with higher odds of cholera (OR = 2.8; 95% CI = 2.1–3.7) with a heterogeneity of *I*^2^ = 45%,^23,26,28,34,35,42,49,57,58,61,65,69^ and safe water transport and storage (*N* = 22) was significantly associated with lower odds of cholera (OR = 0.55, 95% CI = 0.39–0.80) with a heterogeneity of *I*^2^ = 57% (Table 2, Figure 5).^23,25,28,34,46,49,52,59,69^

In the sensitivity analysis, unsafe water storage and transport became insignificantly associated with cholera when only studies with low- and medium-risk bias using the WHO case definition (*N* = 5) were included in the analysis (OR = 1.6, 95% CI = 0.63–4.0) (Supplemental Table 2).

### Sanitation.

Sanitation facilities and proper waste disposal separate feces from the environment. Sanitation facilities were classified based on their description in the articles into the four subcategories of the JMP sanitation ladder: open defecation, unimproved sanitation, shared sanitation, and improved sanitation.^17^ However, please note that for the time during which most of the included studies were conducted, the JMP defined two categories: improved and unimproved sanitation.^70^ Open defecation included cases where feces are disposed in fields, water, and other open spaces and unimproved sanitation includes disposing feces in latrines without a platform, hanging latrines, or bucket latrines. Open defecation and unimproved sanitation do not create a barrier between feces and humans, and were predicted risk factors. Shared sanitation may consist of facilities that adequately separate feces from the environment but are used by two or more households. These facilities are considered unimproved and were predicted risk factors. Improved sanitation facilities ensure separation of feces from the environment, and because this should provide a barrier against fecal–oral transmission, they are predictive protective factors.^21,22^

**Figure 6.**
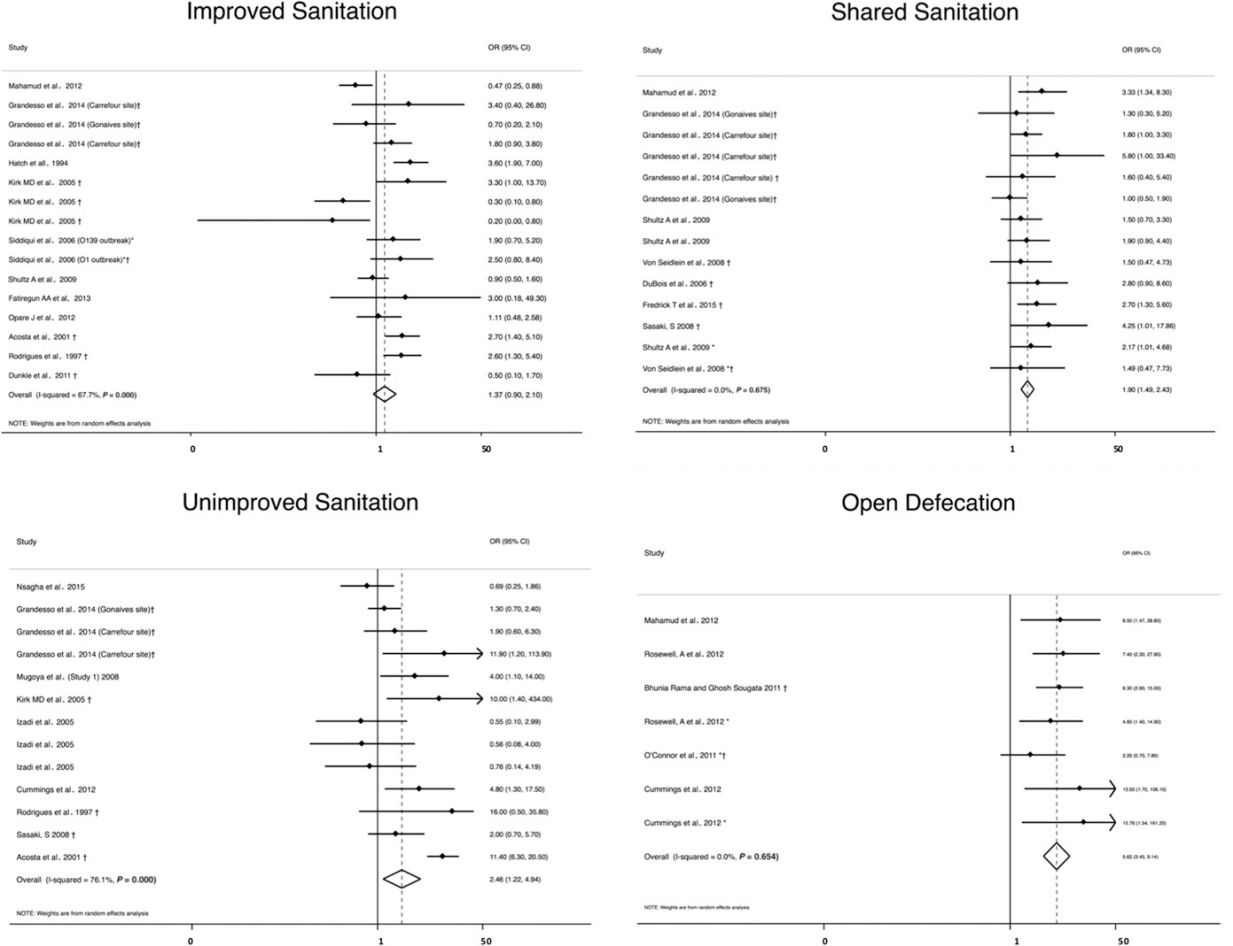
Meta-analysis of the association between sanitation and cholera, including improved and unimproved sanitation, shared sanitation, and open defecation. *Odds ratio (OR) reported from multivariate analysis. †Study used the WHO case definition for cholera.

Improved sanitation (*N* = 16) was not significantly associated with cholera (OR = 1.4, 95% CI = 0.90–2.1, *I*^2^ = 68%).^29,30,32,35,42,46,47,66,69,71,72^ All three predicted risk factors were associated with higher odds of cholera (Table 2, Figure 6). Open defecation displayed the highest OR (*N* = 7, OR = 5.6, 95% CI = 3.5–9.1) with a heterogeneity of *I*^2^ = 0%,^25,28,42,45,61^ followed by unimproved sanitation (*N* = 13, OR = 2.5, 95% CI = 1.2–4.9) with a heterogeneity of *I*^2^ = 76%,^30,35,40,43,46,51,58,61,66^ and shared sanitation (*N* = 14, OR = 1.9, 95% CI = 1.5–2.4) with a heterogeneity of *I*^2^ = 0%.^33,37,42,51,64,66,69^

In the sensitivity analysis, the association between unimproved sanitation and cholera became insignificant when only studies with low and medium bias risk (*N* = 6) were included (OR = 1.9, 95% CI = 0.96–4.0) (Supplemental Table 2).

### Hygiene.

Hygiene includes behaviors that promote cleanliness such as handwashing with soap and water.^21,22^ Signs of good hygiene, including self-reported behaviors or the observation of hygiene materials such as soap and a handwashing area, were predicted protective factors because hygiene creates a barrier between fecal material and the new host (Figure 1).^73,74^ Reporting a lack of hygiene such as no handwashing or handwashing with no soap and water facilitates the spread of disease and is a predicted risk factor.

**Figure 7.**
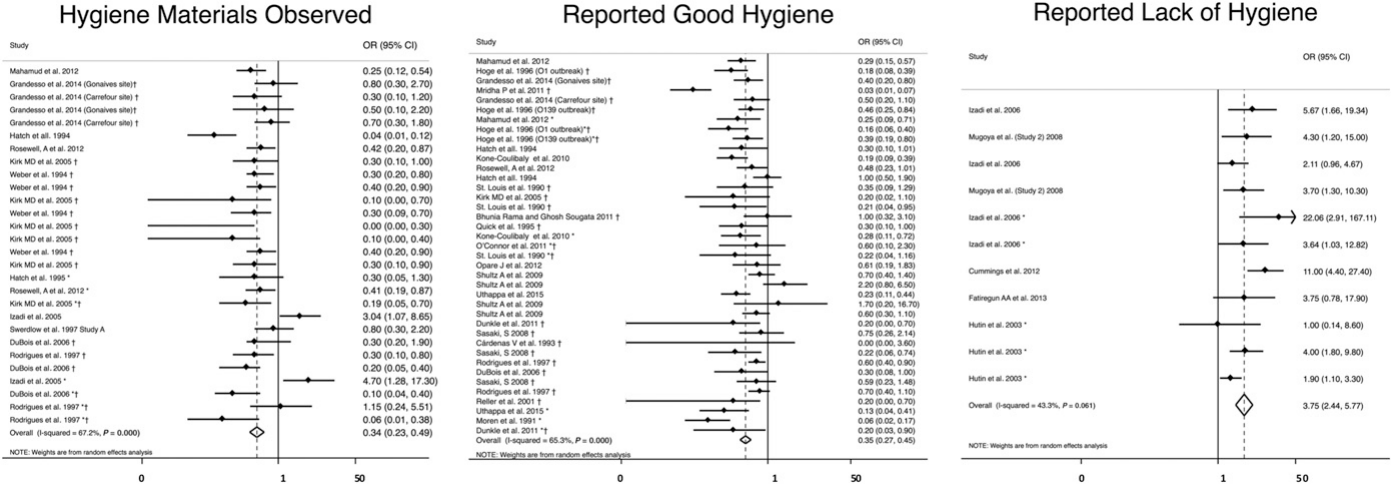
Meta-analysis of the association between hygiene and cholera, including reported good hygiene, reported lack of hygiene, and hygiene materials observed. *Odds ratio (OR) reported from multivariate analysis. †Study used the WHO case definition for cholera.

Lack of hygiene (*N* = 11) was significantly associated with higher odds of cholera (OR = 3.8, 95% CI = 2.4–5.8) with a heterogeneity of *I*^2^ = 43%,^39,58,61,72,75^ whereas self-reported good hygiene (*N* = 39, OR = 0.35, 95% CI = 0.27–0.45)^24,25,27,29,31,32,34,35,42,45,46,48,50,51,56,57,59,64,66,69,71^ and observed good hygiene (*N* = 28, OR = 0.34, 95% CI = 0.23–0.49)^35,40,42,45,46,49,60,64,66,71^ were both significantly associated with lower odds of cholera with a heterogeneity of *I*^2^ = 65% and *I*^2^ = 67%, respectively (Table 2, Figure 7).

There were no changes in direction of association or significance in the sensitivity analysis (Supplemental Table 2).

## DISCUSSION

A systematic review and meta-analysis was conducted to determine the relationship between WASH exposures and cholera. Overall, 47 articles describing 51 case–control studies were included in the review. Water, sanitation, and hygiene exposures were grouped into eight predicted risk factors and seven predicted protective factors. All eight predicted risk factors were associated with increased odds of cholera (OR = 1.9–5.6), with heterogeneity ranging from an *I*^2^ of 0.0–91.8%. Overall, five of seven predicted protective factors were associated with lower odds of cholera transmission (OR = 0.4–1.4), and had higher levels of heterogeneity ranging from *I*^2^ 56.8% to 90.6%. Although the magnitude of associations was similar among risk factors and among protective factors, improved hygiene demonstrated the greatest reduction in the odds of cholera (OR = 0.34 for observed good hygiene), whereas open defecation most increased the odds of cholera (OR = 5.6). Among the predicted protective factors, neither improved water source nor improved sanitation was associated with cholera. In five types of sensitivity analysis, three of 70 summary associations (40%) changed significance from the original analysis, indicating that the results are robust. These results highlight 1) the consistency in association of risk factors with cholera, adding support to current knowledge on the mechanisms of cholera transmission, 2) the inconsistency in the association of cholera with protective factors compared with risk factors, 3) the unexpected finding that no sanitation factor or improved water source (with the exception of bottled water) was significantly protective, and 4) the need for more specific reporting of intervention details in case–control studies.

Summary ORs for each factor suggest that overall, predicted risk factors are a risk for cholera transmission. These data underscore the importance of WASH factors for the transmission of cholera, with all suspected pathways showing an association with cholera across a diversity of contexts. Although the most important transmission pathways may differ by context and an assessment of these pathways may be undertaken during an outbreak to target interventions, these results show that even pathways that are less commonly focused on during outbreaks (e.g., sanitation) are associated with cholera and, therefore, broad programming such as frequently implemented “WASH packages” may be indicated.^6^

Results were less consistent for predicted protective factors. Only five of seven protective factors showed a significant negative association with cholera, and all protective factors had wide CIs and statistically significant heterogeneity. Our findings suggest that risk factors are consistently risky, and factors expected to interrupt cholera transmission have the potential to do so, but are not always effective. This is likely partly because cholera is transmitted via multiple pathways (Figure 1) such that removing one source of contamination may not effectively prevent disease, whereas the introduction of contamination through a single pathway can effectively cause disease. Our results are consistent with literature which shows that individual interventions can have differing levels of effectiveness in different contexts and that the efficacy of an intervention under ideal conditions and its effectiveness for preventing disease transmission in given context are different.^76–78^ Furthermore, access to “improved” WASH does not necessarily imply that exposures are microbiologically safe or consistently used properly. For example, piped water is an “improved” source that is also sometimes seen as the source of cholera outbreaks, and even if an intervention such as water treatment is regularly used, cholera can be transmitted when a mistake is made in dosing or treatment is forgotten. We suspect that cultural differences around WASH practices, eating, and drinking also contribute this variation, in addition to differences in the exposures themselves. Effectiveness depends on, among other factors, a good program design that targets pathways contributing to disease transmission and accounts for beneficiary preferences to ensure correct and consistent use.^6,79^ Our findings emphasize that cholera risk lies along many pathways and proper implementation and strong uptake of WASH interventions are critical components to success.

The one WASH group in which no factor was protective was sanitation; all four factors were associated with increased cholera. These factors were adopted from the 2008 JMP sanitation ladder, which provides a detailed framework for assessing sanitation; rather than considering sanitation as simply improved or unimproved, sanitation facilities are classified into open defection, unimproved facilities, shared facilities, and improved facilities.^20,70^ Although we expect containment of feces to reduce disease risk, the relationship between these classifications and disease risk is uncertain and lacks a foundation in evidence.^80,81^ Although no sanitation factor was consistently protective, the magnitude of the association between sanitation factors and cholera decreased as households climbed the sanitation ladder from open defecation to improved sanitation, suggesting that the ladder has a reasonable epidemiological foundation. The current sanitation ladder does not include treatment of waste, which has been shown to reduce disease transmission.^82,83^ The current Sustainable Development Goals (SDGs) are considering waste treatment at a global scale.^84^ Although no sanitation factor was associated with reduced odds of cholera, these results do not suggest that sanitation is unimportant; rather, they suggest that this pathway may be more important for cholera transmission than previously thought and further research is needed on sanitation and cholera.

The other predicted protective factor that did not show an association with cholera was improved water source. Although improved sources should protect water from outside contamination, they do not always provide safe water. The JMP conducted a “Rapid Assessment of Drinking Water Quality,” which found that although improved sources sometime met the WHO drinking-water quality standards, they often did not; in one case, only 34% of samples from improved water sources met safety guidelines.^85^ Improved water sources can become contaminated for many reasons. For example, piped water supply may be intermittent, allowing for backflow and intrusion of contaminated water, or rainwater collection containers may be contaminated. This variability in quality of water from improved sources is seen in the lack of association between improved source and cholera. In some case–control studies, it even appeared that improved water sources were the source of an outbreak, especially from failing piped water systems.^25,31,33,86^ The SDGs acknowledge that improved water sources do not always function as intended and are focusing on the provision of safe water.^84^ We recommend responders follow the SDG guidelines and confirm whether improved sources are providing water that is safe to drink.

As described previously, good implementation and consistent uptake are critical for WASH interventions to interrupt disease transmission. One of the challenges in completing this review was that WASH interventions are often incompletely described in case–control studies, making it difficult to assess what the WASH intervention was or why it may or may not have been successful. It has been documented that the success or failure of interventions often occurs at least partly through pathways that are not considered for primary assessments (e.g., provision of water makes household money available for purchasing more soap).^87^ Knowledge and attitudes toward WASH interventions and standard practices may also impact intervention effectiveness, but these factors were rarely reported and are, therefore, not covered in this review. We suspect that the high degree of heterogeneity seen among protective factors is due to undescribed differences, rather than the innate ability of an intervention to provide efficacious protection. Considering this, we recommend that reports of outbreaks should include detailed information on the design and implementation of interventions, so that factors leading to success or failure can be directly assessed and implemented in the future, to prevent disease transmission.

Our study had several limitations. Our analysis was limited to peer-reviewed articles published in English; more data are likely available in other languages and gray literature. Because we used data from studies in peer-reviewed articles, it is possible that publication bias led to underreporting of null findings; this would affect results by making summary ORs more extreme. Furthermore, most of the studies were classified as “high risk of bias.” Although in most cases a sensitivity analysis in which high risk of bias studies was removed did not change the result, we did find that there was no longer an association between cholera and water source and management when high-risk studies were removed. This suggests that although in general the results are relatively robust, low-quality studies could falsely influence associations and caution should be used in interpreting all results. Although every effort was made to place factors in the correct category, lack of intervention detail may have led to misclassification bias. Data were self-reported, which may have also resulted in misclassification.

Included studies also lacked data that would have enabled us to better describe the context of the observed associations. Ideally, we would be able to perform a multivariate meta-analysis that would account for effect modification when factors coexist. However, although multiple exposures were often reported in a single study, studies were of low enough quality with missing data on exposures and interstudy correlations that we did not feel it was possible to construct a robust multivariate analysis. We suspect that effect modification within an outbreak is another piece of context that might explain the variability observed in interventions. Although we have data on outbreak setting (i.e., urban and rural), the split was too uneven to allow us to draw robust conclusions. Future research might also be designed to include a theory of behavior change and incorporate factors such as exposure to cholera risk messaging. Last, although we suspect that methods and quality of implementation of interventions would have an impact on their effectiveness, the included studies did not provide sufficient detail to assess this and include in analysis. Despite these limitations, we feel that these results add value and highlight ways future case–control studies could collect detailed intervention data to address these limitations.

Overall, our results support the conclusion that risk factors allowing for disease transmission are associated with greater odds of cholera during an outbreak and should be addressed to limit disease transmission, and protective factors expected to provide a barrier to transmission are associated with lower odds of cholera. However, the effect of predicted protective factors was inconsistent, as interventions may not have the intended effect if they are not implemented properly. We recommend that interventions delivered during cholera outbreaks should be implemented in a way that promote correct and consistent use, and future case–control studies should detail the design and implementation characteristics of WASH interventions so that factors leading to success or failure can be more directly assessed and implemented in the future to prevent disease transmission.

## Supplementary Material

Supplemental tables
