# Supplementary material for: A Systematic Review and Meta-Analysis of the Association between Water, Sanitation, and Hygiene Exposures and Cholera in Case–Control Studies
Source: Am J Trop Med Hyg. 2018 Jul 2;99(2):534–45. doi: 10.4269/ajtmh.17-0897 (PMC6090371; doi:10.4269/ajtmh.17-0897)
Supplement: Supplementary file 1 [file tpmd170897.SD1.pdf]

Table S1. List of manuscripts and case-control studies included

**Author**	**Year**	**Country**	**Outbreak Setting**	**Outbreak year**	**WHO Cholera Definition**	**Cases**	**Controls**	**Summary Bias Score**
Acosta et al	2001	Tanzania	Rural	1997	Yes	180	360	HIGH
Beatty et al	2004	Republic of Marshalls Islands	Urban	2000	Yes	53	104	LOW
Rama and Sougata	2011	India	Rural	2009	Yes	57	171	MEDIUM
Birmingham et al	1997	Burundi	Urban	1992	Yes	56	112	HIGH
Cummings et al	2012	Uganda	Rural	2010	No	99	99	HIGH
DuBois et al	2006	Zambia	Urban	2003-2004	Yes	71	71	HIGH
Dunkle et al	2011	Haiti	Urban	2010	Yes	53	106	HIGH
Fredrick T et al	2015	India	Urban	2012	Yes	66	88	HIGH
Grandesso et al	2014	Haiti	Urban	2010	Yes	90	180	LOW
Grandesso et al	2014	Haiti	Urban	2010	Yes	90	180	LOW
Hatch et all	1994	Malawi	Refugee camp	1988	No	48	441	MEDIUM
Hoge et al	1996	Thailand	Urban	1993	Yes	105	210	LOW
Hoge et al	1996	Thailand	Urban	1993	Yes	103	206	LOW
Izadi et al	2006	Iran	Rural	2004	No	85	85	MEDIUM
Koo D et al	1996	Guatemala	Urban	1991	Yes	26	52	HIGH
Mahamud et al	2012	Kenya	Refugee camp	2009	No	93	93	LOW
Moren et al	1991	Malawi	Refugee camp	1988	No	51	51	HIGH
Mridha P et al	2011	India	Urban	2010	Yes	60	120	LOW
Mugoya et al	2008	Kenya		2005	No	34	68	MEDIUM

Mugoya et al	2008	Kenya		2005	No	34	68	MEDIUM
Mujica et al	1994	Peru	Urban	1991	No	50	100	HIGH
Nguyen et al	2014	Sierra Leone	Urban	2012	Yes	49	98	LOW
Nsagha et al	2015	Cameroon	Rural	2011	No	34	101	LOW
O'Connor et al	2011	Haiti	Rural	2010	Yes	49	98	MEDIUM
Quick et al	1995	El Salvador	Rural	1991	Yes	24	48	MEDIUM
Ries et al	1992	Peru	Urban	1991	Yes	50	100	HIGH
Rodrigues et al	1997	Guinea- Bissau	Urban	1994-1995	Yes	206	206	HIGH
Rodrigues et al	2000	Guinea-Bissau	Urban	1996	Yes	221	221	MEDIUM
Rosewell, et al	2012	Papua New Guinea	Urban	2009	No	54	122	MEDIUM
Sasaki, S	2008	Zambia	Urban	2003-2004	Yes	40	80	HIGH
Shapiro et al	1999	Kenya	Rural	1997	Yes	103	113	HIGH
Shultz et al	2009	Kenya	Refugee camp	2005	No	90	170	HIGH
Siddiqui et al	2006	Pakistan	Rural	2002	No	30	194	HIGH
Siddiqui et al	2006	Pakistan	Rural	2003	Yes	37	71	HIGH
Swerdlow et al	1997	Malawi	Refugee camp	1990	No	50	50	HIGH
Swerdlow et al	1997	Malawi	Refugee camp	1990	No	47	137	HIGH
Swerdlow et al	1992	Peru	Urban	1991	No	46	65	HIGH
Ujjiga et al	2015	South Sudan		2014	No	134	134	HIGH
Von Seidlein et al	2008	Mozambique	Urban		Yes	40	127	HIGH
Weber et al	1994	Ecuador	Urban	1991	Yes	63	126	MEDIUM
Cárdenas V et al	1993	Colombia	Urban	1992	Yes	32	38	HIGH
De Guzman et al	2015	Philippines	Urban and Rural	2012	No	55	110	HIGH
Fatiregun et al	2013	Nigeria	Urban	2010	No	22	44	HIGH
Hutin et al	2003	Nigeria	Urban		No	102	77	HIGH
Kirk MD et al	2005	Federated States of Micronesia	Urban	2000	Yes	53	53	MEDIUM

Kone-Coulibaly et al	2010	Zimbabwe	Urban	2008	No	140	140	MEDIUM
Opare J et al	2012	Ghana	Urban	2010	No	39	78	HIGH
St. Louis et al	1990	Guinea	Urban	1986	Yes	35	70	MEDIUM
Uthappa et al	2015	India	Rural	2013	No	138	138	HIGH
Izadi et al	2005	Iran	Rural	2003	No	20	89	HIGH
Reller et al	2001	Madagascar	Urban	2000	Yes	37	74	HIGH

Table S2. Summary odds ratios and heterogeneity analysis for WASH exposures in cholera outbreaks

	E(n)	M(n)	OR (95% CI)	I^2	E(n)	M(n)	OR (95% CI)	I^2
	**Improved Water Source**				**Unimproved Water Source**			
**All**	28	18	1.1 (0.54 – 2.2)	91%**	38	23	3.4 (2.5 – 4.7)	71%**
**Low & Med Bias**	11	6	0.68 (0.17 – 2.7)	94%**	14	10	2.5 (1.3 – 5.1)	82%**
**WHO Def**	21	13	1.7 (0.86 – 3.4)	86%**	17	11	3.7 (2.3 – 5.8)	56%**
**Low Bias**	3	1	-	-	7	6	2.5 (1.0-6.1)	72%
**Low Bias & WHO Def**	3	1	-	-	4	2	-	-
**Low & Med & WHO Def**	9	5	1.5 (0.38 – 5.5)	88%**	7	4	2.4 (0.78 – 7.7)	75%**
	**Bottled Water**				**Surface Water Contact**			
**All**	7	5	0.35 (0.13 - 0.96)	77%**	10	7	2.3 (1.1 – 4.8)	92%**
**Low & Med Bias**	1	1	-	-	4	3	-	-
**WHO Def**	4	3	-	-	6	4	3.0 (1.0 – 8.7)	95%**
**Low Bias**	1	1	-	-	0	0	-	-
**Low Bias & WHO Def**	1	1	-	-	0	0	-	-
**Low & Med & WHO Def**	1	1	-	-	3	2	-	-
	**Treated Water**				**Untreated Water**			
**All**	40	14	0.44 (0.35 - 0.56)	61%**	34	18	3.5 (2.8 – 4.4)	48%**
**Low & Med Bias**	22	6	0.47 (0.46 - 0.60)	46%**	20	8	4.1 (2.9 – 5.9)	55%**
**WHO Def**	35	11	0.52 (0.42 - 0.64)	49%**	27	14	3.6 (2.7 – 4.8)	56%**
**Low Bias**	9	3	0.61 (0.46 – 0.82)	21%	11	3	3.6 (2.6 – 5.0)	14%
**Low Bias & WHO Def**	8	2	0.65 (0.49 – 0.85)	12%	11	3	3.6 (2.6 – 5.0)	14%
**Low & Med & WHO Def**	21	5	0.48 (0.37 - 0.62)	47%**	18	7	4.3 (2.9 – 6.2)	60%**
	**Safe Storage and Transport**				**Unsafe Storage and Transport**			
**All**	22	9	0.55 (0.39 - 0.80)	57%**	22	13	2.8 (2.1 - 3.7)	45%*
**Low & Med Bias**	16	5	0.42 (0.28 - 0.63)	46%*	10	6	2.6 (1.4 – 4.6)	70%**
**WHO Def**	18	7	0.51 (0.32 - 0.80)	62%**	11	7	2.4 (1.5 - 3.7)	65%**
**Low Bias**	11	1	-	-	4	2	-	-
**Low Bias & WHO Def**	11	1	-	-	4	2	-	-
**Low & Med & WHO Def**	16	5	0.42 (0.28 - 0.63)	46%*	5	4	1.6 (0.63 – 4.0)	80%**
	**Improved Sanitation**				**Open defecation**			
**All**	16	11	1.4 (0.90 – 2.1)	68%**	7	5	5.6 (3.5 - 9.1)	0.0%
**Low & Med Bias**	10	5	1.3 (0.69 – 2.5)	74%**	5	4	5.1 (3.0 - 8.5)	0.0%
**WHO Def**	10	6	1.4 (0.83 – 2.5)	60%**	2	2	-	-
**Low Bias**	4	2	-	-	1	1	-	-
**Low Bias & WHO Def**	3	1	-	-	0	0	-	-
**Low & Med & WHO Def**	7	3	1.2 (0.58-2.7)	59%*	2	2	-	-
	**Self-reported good hygiene**				**Unimproved Sanitation**			
**All**	39	22	0.35 (0.27 - 0.45)	65%**	13	9	2.5 (1.2 – 4.9)	76%**
**Low & Med Bias**	21	12	0.31 (0.22 – 0.43)	60%**	6	4	1.9 (0.96 – 4.0)	49%
**WHO Def**	24	14	0.33 (0.23 – 0.46)	62%**	7	5	4.3 (1.6 – 11)	81%**
**Low Bias**	9	3	0.25 (0.15 – 0.41)	72%**	4	2	-	-
**Low Bias & WHO Def**	7	3	0.24 (0.12 – 0.45)	79%**	3	1	-	-
**Low & Med & WHO Def**	14	8	0.28 (0.18 – 0.44)	63%**	4	2	-	-
	**Observation of hygiene materials**				**Shared Sanitation**			
**All**	28	11	0.34 (0.23 - 0.49)	67%**	14	7	1.9 (1.5 - 2.4)	0.0%
**Low & Med Bias**	19	7	0.30 (0.21 – 0.42)	50%**	6	2	1.8 (1.1-2.7)	24%
**WHO Def**	20	5	0.29 (0.20 – 0.42)	45%*	10	5	1.8 (1.3-2.5)	0.0%
**Low Bias**	5	2	-	-	6	2	-	-
**Low Bias & WHO Def**	4	1	-	-	5	1	-	-
**Low & Med & WHO Def**	14	3	0.32 (0.21 – 0.49)	45%*	5	1	-	-
					**Self-reported lack of hygiene**			
**All**					11	5	3.8 (2.4 – 5.8)	43%
**Low & Med Bias**					6	2	3.7 (2.3-6.0)	8.0%
**WHO Def**					0	0	-	-

**Low Bias**	0	0	-	-
**Low Bias & WHO Def**	0	0	-	-
**Low & Med & WHO Def**	0	0	-	-

E (N) = number of factors with OR reported; M (N) = number of manuscripts; *significant at the $p<0.05$ level; **significant at the $p<0.01$ level
